# Assessment of myocardial fibrosis by late gadolinium enhancement imaging and biomarkers of collagen metabolism in chronic rheumatic mitral regurgitation 

**DOI:** 10.5830/CVJA-2018-002

**Published:** 2018

**Authors:** Meel Ruchika, Nethononda Richard, Libhaber Elena, Dix-Peek Therese, Peters Ferande, Essop Mohammed

**Affiliations:** Division of Cardiology, Chris Hani Baragwanath Academic Hospital and University of the Witwatersrand, Johannesburg, South Africa; Division of Cardiology, Chris Hani Baragwanath Academic Hospital and University of the Witwatersrand, Johannesburg, South Africa; Division of Cardiology, Chris Hani Baragwanath Academic Hospital and University of the Witwatersrand, Johannesburg, South Africa; Division of Cardiology, Chris Hani Baragwanath Academic Hospital and University of the Witwatersrand, Johannesburg, South Africa; Division of Cardiology, Chris Hani Baragwanath Academic Hospital and University of the Witwatersrand, Johannesburg, South Africa; Division of Cardiology, Chris Hani Baragwanath Academic Hospital and University of the Witwatersrand, Johannesburg, South Africa

**Keywords:** chronic rheumatic mitral regurgitation, cardiac magnetic resonance, late gadolinium enhancement, biomarkers

## Abstract

**Background:**

In chronic rheumatic mitral regurgitation (CRMR), involvement of the myocardium in the rheumatic process has been controversial. Therefore, we sought to study the presence of fibrosis using late gadolinium enhancement cardiac magnetic resonance imaging (LGE–CMR) and biomarkers of collagen turnover in CRMR.

**Methods:**

Twenty–two patients with CRMR underwent CMR and echocardiography. Serum concentrations of matrix metalloproteinase– 1 (MMP–1), tissue inhibitor of MMP–1 (TIMP– 1), MMP–1–to–TIMP–1 ratio, procollagen III N–terminal pro–peptide (PIIINP) and procollagen type IC peptide (PIP) were measured.

**Results:**

Four patients had fibrosis on LGE–CMR. PIP and PIIINP concentrations were similar to those of the controls, however MMP–1 concentration was increased compared to that of the controls (log MMP–1 3.5 ± 0.7 vs 2.7 ± 0.9, p = 0.02). There was increased MMP–1 activity as the MMP–1–to– TIMP–1 ratio was higher in CRMR patients compared to the controls (–1.2 ± 0.6 vs –2.1 ± 0.89, p = 0.002).

**Conclusion:**

Myocardial fibrosis was rare in CRMR patients. CRMR is likely a disease characterised by the predominance of collagen degradation rather than increased synthesis and myocardial fibrosis.

Myocardial fibrosis can be reliably detected non–invasively using late gadolinium enhancement (LGE–CMR) or contrast enhancement cardiac magnetic resonance (CE–CMR) imaging.^1^ CE–CMR is a useful non–invasive correlate of myocardial fibrosis on histology.^2^ Fibrosis represents an end–stage process in various cardiac conditions, irrespective of aetiology and denotes adverse outcomes.^3^ Limited recent studies have shown the value of CMR in valvular heart disease, such as degenerative MR and aortic stenosis, in predicting the prognosis based on the presence of fibrosis.^2^,^4^,^5^

Studies pertaining to the possible involvement of the left ventricle (LV) in the rheumatic process have yielded equivocal results.^6^ In chronic rheumatic mitral regurgitation (CRMR) there may be involvement of the LV in the rheumatic process, especially in the posterobasal region of the LV.^7^–^9^ Sepulveda et al. reported diffuse, mesocardial and heterogenous enhancement of the myocardium in acute rheumatic fever using LGE.^10^ The possible resultant fibrosis may therefore be studied by LGE and have prognostic value similar to that in degenerative MR. Furthermore, data concerning biomarkers of collagen degradation and formation in MR are limited and mostly comprise animal studies in degenerative MR.^11^,^12^

In a recent study in rheumatic MR, an increase in biomarkers of collagen synthesis and degradation was reported.^13^ Biomarkers of collagen turnover may serve as non–invasive tools for identification of myocardial remodelling and add an incremental value in risk stratification for surgery or institution of aggressive medical treatment at an early stage.^14^–^16^

Procollagen III N–terminal pro–peptide (PIIINP) and procollagen IC peptide (PIP) are released into the circulation during collagen synthesis, while the turnover of collagen is controlled by matrix metalloproteinases (MMPs) and their inhibitors, the tissue inhibitors of metalloproteinases (TIMPs).^14^ These markers are therefore an excellent model to study collagen turnover. Therefore we sought to assess the presence of LV fibrosis in CRMR using cardiac MRI and biomarkers of collagen degradation and synthesis.

## Methods

This study was part of a prospective, cross–sectional study at Chris Hani Baragwanath Academic Hospital. Patients were enrolled from January to October 2014. All patients were screened and those deemed to have moderate or severe CRMR were referred for possible inclusion in the study.

The inclusion criteria were patients aged 18 years or older with echocardiographic features of moderate or severe CRMR. Patients were excluded if they had co–morbidities, significant aortic valve disease, concurrent mitral stenosis with a valve area of less than 2.0 cm^2^, documented ischaemic heart disease, pre–existing non–valvular cardiomyopathy, prior cardiac surgery, congenital or pericardial disease, pregnancy, severe anaemia (haemoglobin < 10 g/dl), presence of a pacemaker or defibrillator, claustrophobia, renal dysfunction (estimated glomerular filtration rate, eGFR < 60 ml/min), or refusal to undergo CMR.

A final number of 91 patients with presumed CRMR underwent clinical evaluation, resting electrocardiogram and detailed echocardiographic assessments according to a pre–determined protocol. Of these 91 patients with CRMR, 69 were excluded due to the following: co–morbidities (human immunodeficiency virus: n = 22; hypertension: n = 44; diabetes mellitus: n = 3; atrial fibrillation: n = 4; anaemia: n = 3; renal dysfunction: n = 3; and inadequate image quality: n = 5).

The final sample comprised 22 patients. Fourteen age– and gender–matched controls were also enrolled for the biomarker arm of the study. A tolerance of five years was allowed for age matching.The baseline clinical characteristics of these individuals were recorded and they subsequently underwent comprehensive echocardiography and CMR imaging.

The study was approved by the University of the Witwatersrand ethics committee. It was conducted in accordance with the principles outlined in the Declaration of Helsinki.

Transthoracic echocardiography was performed on all patients in the left lateral position by experienced sonographers using a S5–1 transducer on a Philips iE33 system (Amsterdam, the Netherlands). Images were obtained according to a standardised protocol. The data were transferred and analysed off–line using the Xcelera workstation.

All linear chamber measurements were performed according to the American Society of Echocardiography (ASE) chamber guidelines.^17^ Measurements relating to LV diastolic function were performed in accordance with the ASE guidelines on diastolic function, and included pulse–wave Doppler at the mitral tips and tissue Doppler of both medial and lateral mitral annuli.^18^

MR was considered rheumatic in aetiology when the morphology of the valve satisfied the World Heart Federation (WHF) criteria for the diagnosis of chronic rheumatic heart disease (RHD).^19^ MR severity was assessed using qualitative, semi–quantitative and quantitative methods as per the ASE and European Society of Cardiology.^20^,^21^ In equivocal cases, the echocardiographic data were integrated with the clinical evaluation by an experienced cardiologist to distinguish moderate from severe MR.

For biomarker analysis, peripheral venous blood samples were drawn from 14 controls and 22 chronic rheumatic heart disease subjects at the time of echocardiographic examination. Samples were collected in a serum separator tube and allowed a clotting time of 30 min before centrifuging for 15 min at 1 000 g and the serum was stored at –80°C before analysis in a single batch.

Enzyme–linked immunosorbent assays (ELISA) were used to determine the serum concentration of PIIINP (USCN Life Science Inc/Cloud–Clone Corp, Wuhan, China), and PIP (Clontech,Takara Bio Inc, Japan) was determined by ELISA using the USCN kit according to the manufacturer’s instructions. The minimum detectable dose of PIIINP is typically less than 25.9 pg/ml and the minimum detectable dose of PIP is typically less than 10 ng/ml. Optical density of the analytes was measured at 450 nm on a microplate reader (Elx800, Biotek, USA) and concentrations were determined using a 5–PL algorithm.

Multi–analyte analysis of the matrix metalloproteinase 1 (MMP–1) and tissue inhibitor of metalloproteinase 1 (TIMP– 1) was performed using the magnetic luminex screening assay (RnD systems, Minneapolis, USA) on a Bio–Plex 200 (Bio–Rad, CA, USA) according to the manufacturer’s instructions. The minimum detectable dose of MMP–1 is typically less than 2.7 pg/ml, and for TIMP–1, less than 3.42 pg/ml. Since the actual activity of MMP–1 depends on the balance between active enzyme and inhibitor (i.e. TIMP–1), the serum MMP–1/TIMP–1 ratio was considered an index of MMP–1 activity.

CMR studies were performed on a 1.5–Tesla scanner (Siemens Healthcare, Erlagen, Germany) using a six–channel phasedarray body coil. The images were obtained during patient expiratory breath–hold for approximately eight seconds and were prospectively ECG gated.^22^ LV volumes and mass were acquired in line with standard cardiovascular MRI protocols (1.5–T magnetom Avanto; Siemens Healthcare, Erlangen, Germany). Steady–state free–precession imaging (echo times 1.5/3.0 ms, flip angle 60°, temporal resolution 45 ms, slice thickness 7 mm, 3–mm gap, matrix size 256 × 256 mm, field of view 380 × 309 mm) were performed to obtain long–axis cines and a contiguous stack of short–axis cines for assessment of LV volumes, mass and ejection fraction, as previously described.^23^

Ten minutes after the injection of 0.2 mmol/kg gadoliniumbased contrast agent (Magnevist, Schering, Berlin, Germany), LGE–CMR images were acquired in the same long– and shortaxis position, and used in the cine imaging.^5^ Inversion recovery times varying from 200–350 ms were used to null the signal from the intact myocardium.

Images were analysed by an independent experienced reader (RN), blinded to the echocardiographic results, with Argus software (version 2002B, Siemens Medical Solutions, Erlangen), as previously described.^24^ The assessment of cardiac function and chamber sizes were performed in standard views in the long-axis (horizontal and vertical) and short–axis planes. Ejection fraction for the LV was assessed with the following formula:

Ejection fraction = end-diastolic volume - end-systolic volume/end-diastolic volume

LV volumes and EF were obtained by semi–automatic tracing of contours on the short–axis images in end–diastole and end–systole, with manual corrections when required.^25^ Myocardial fibrosis was defined as a region of LGE with signal enhancement greater than the signal intensity of non–enhanced myocardium.^1^

## Statistical analysis

Statistical analysis was performed with Statistica version 12.5, series 0414 for Windows. Continuous variables are expressed as means ± SD or medians (IQRs). Categorical data are expressed as a percentage. The differences for continuous variables were calculated using the Student’s t–test or Mann–Whitney U–test when the distribution was non–normal. Chi–squared and Fisher’s exact tests were used to calculate the difference for categorical data for independent samples. Pearson’s and Spearman’s correlation coefficient were used to calculate correlations depending on whether data were normally or non–normally distributed. Biomarker levels (TIMP–1, MMP–1 and MMP–1/TIMP–1 ratio) were log transformed before analysis when distribution was not normal. A p–value < 0.05 was considered statistically significant.

## Results

The mean age of patients was 36.3 ± 13.9 years, with 81% female (Table 1). All the patients had isolated moderate or severe CRMR and no co–morbidities. Ten patients were in New York heart association (NYHA) functional class I, the remainder were NYHA functional class II. Four patients were on medical treatment with diuretics (furosemide) and anti–remodelling therapy (spironolactone, carvedilol, enalapril) for previous heart failure secondary to MR. Eight patients were on diuretics alone.

**Table 1:** Clinical and echocardiographic characteristics of the study patients and controls

*Variable*	*Study group (n = 22)*	*Control (n = 14)*	*p-value*
Clinical parameters
Age (years)	36.3 ± 13.9	40.3 ± 14.2	0.40
Gender (F:M)	18:4	10:4	0.36
SBP (mmHg)	123.2 ± 9.5	122.9 ± 5.1	0.91
DBP (mmHg)	77.2 ± 6.4	74.6 ± 12.3	0.34
Pulse (beats/min)	74.6 ± 13.1	75.5 ± 13.3	0.55
Body mass index (kg/m^2^)	24.8 ± 4.7	28 ± 5.7	0.06
Body surface area (m^2^)	1.6 ± 0.2	1.7 ± 0.2	0.24
Echocardiographic parameters			
LVEDD (mm)	56.2 ± 7.4	42.2 ± 6.1	< 0.001
LVESD (mm)	41.5 ± 8.6	26.7 ± 4.0	< 0.001
EDVi (ml/m^2^)†	90.4 (71.5–103.8)	43.2 (35.2–43.2)	< 0.001
ESVi (ml/m^2^)†	39.6 ± 19.6	15.3 ± 4.6	0.001
LVEF (%)	59.8 ± 10.6	60.6 ± 17.1	0.5
LV mass index (g/m^2^)†	100.1 ± 33.8	61.4 ± 18.7	0.004

†Data are presented as median (interquartile range), mean ± SD or %. Values are indexed to BSA.

DBP: diastolic blood pressure; SBP: systolic blood pressure; LV: left ventricle; EDD: end-diastolic diameter; ESD: end-systolic diameter; EDVi: end-diastolic volume indexed; ESVi: end-systolic volume indexed; LVEF: left ventricular ejection fraction.

In this study, LGE was present in four (18%) patients with CRMR (Table 2). A varied pattern of LGE of the LV myocardium was noted. These included (1) transmural LGE in the lateral wall, (2) patchy areas of LGE in the basal septum, mid–septum and basal inferior wall, (3) transmural fibrosis of the inferior wall, and (4) sub–epicardial LGE in one patient. The two patients with transmural involvement had normal coronary angiograms (done as part of their surgical work–up).

**Table 2:** CMR characteristics of the study patients

*CMR characteristics*	*Values*
Regurgitant volume (ml)	47.0 ± 19.9
Regurgitant fraction (%)	49.2 (31.7–56.2)
EDVi (ml/m^2^)†	98.5 (81–111.1)
ESVi (ml/m^2^)†	49.1 ± 36.7
LVEF (%)	58.8 ± 15.1
Moderate MR, n (%)	9 (41)
Severe MR, n (%)	13 (55)

†Data are presented as median (interquartile range), mean ± SD or %. Values are indexed to BSA.

EDVi: end-diastolic volume indexed; ESVi: end-systolic volume indexed; LVEF: left ventricular ejection fraction; MR: mitral regurgitation.

PIIINP and PIP were not elevated in patients compared to controls. PIIINP concentrations were 11.8 (6.9–21.6) vs 15.7 (13.6–18.5) ng/ml (p = 0.09), while PIP levels were 780.4 (727.3–1263.7) vs 1065.1 (589.2–1252.0) μg/ml (p = 0.13) (Table 3). Log MMP–1 was elevated in patients with CRMR compared to the controls (3.45 ± 0.7 vs 2.7 ± 0.9, p = 0.02). There was no difference in log TIMP–1 between CRMR patients and controls (4.6 ± 0.39 vs 4.8 ± 0.30, p = 0.15). The ratio of log MMP–1 to TIMP–1 was increased (–1.2 ± 0.6 vs –2.1 ± 0.89, p = 0.002) in the study patients compared to the controls.

**Table 3:** Biomarkers in the study patients compared to controls

*Biomarkers*	*Study group (n = 22)*	*Control (n = 14)*	*p-value*
PIIINP (ng/ml)	11.8 (6.9-21.6)	15.7 (13.6-18.5)	0.09
Log PIIINP	2.5 ± 0.7	2.7 ± 2.6	0.18
PIP (μg/ml)	780.4 (727.3–1263.7)	1065.1 (589.2–1252.5)	0.13
Log PIP	6.79 ± 0.57	6.8 ± 0.47	0.29
MMP-1 (ng/ml)	37.5 (19.9–59.7)	16.2 (6.53–37.9)	0.3
Log MMP-1	3.45 ± 0.7	2.7 ± 0.9	0.02
TIMP-1 (ng/ml)	95.4 (90.4–140.1)	139.2 (110.3–155.5)	0.1
Log TIMP-1	4.6 ± 0.4	4.8 ± 0.30	0.15
MMP-1/TIMP-1 ratio	0.26 (0.21–0.43)	0.11 (0.07–0.26)	0.08
Log MMP-1/TIMP-1 Ratio	–1.2 ± 0.6	–2.05 ± 0.89	0.002

Data are presented as median (interquartile range), mean ± SD or %.

PIIINP: procollagen III N-terminal pro-peptide; PIP: procollagen type IC peptide; MMP: matrix metalloproteinase; TIMP: tissue inhibitor of matrix metalloproteinase.

## Discussion

The main findings of this study were: fibrosis, as assessed by LGE was uncommon in CRMR; and biomarkers suggestive of collagen degradation (MMP–1, MMP–1/TIMP–1 ratio) were increased in CRMR, but no changes in biomarkers of collagen synthesis (PIP and PIIINP) were noted.

In this study, the majority of patients with CRMR did not have LV myocardial fibrosis on LGE. There are no studies on CRMR to draw comparisons from, but the limited studies done in degenerative MR have shown the presence of fibrosis on LGE in about 30% of patients compared to only 18% in the current study.^4^,^26^ In contrast with our study, biological factors such as advanced age, and co–morbidities such as hypertension and diabetes may have contributed to the higher prevalence of fibrosis in these studies.^4^,^26^ Furthermore, one study used T1 mapping in addition to LGE, and was able to report on microvascular fibrosis, increasing the detection rate of fibrosis in their study.^4^ An alternative explanation for a lack of fibrosis in the majority of patients in this study may be the presence of diffuse fibrosis, which is missed by the LGE technique, as it compares regions of normal myocardium to abnormal myocardium.^1^ Conversely, fibrosis may indeed have been absent, and this is supported by the normal markers of collagen synthesis in this study.

The above hypothesis is further supported on the basis of a study done by Ho et al. in hypertrophic cardiomyopathy patients, where it was noted that a pro–fibrotic state (as assessed by increased biomarkers of synthesis) preceded the development of fibrosis visible on MRI.^27^ The sample size in our study was too small to draw comparisons based on the presence or absence of LGE, or to comment on patterns of enhancement in detail. Interestingly though, LV fibrosis in the four patients was not confined to the posterobasal region, an area noted to be affected more commonly by rheumatic fever.^7^

A higher prevalence of fibrosis is observed commonly in pressure–overload states such as aortic stenosis.^2^ The exact mechanism of greater fibrosis in pressure–overload states compared to volume–overload states remains speculative.^28^ The following reasons have been proposed: (1) a greater supply/ demand mismatch in pressure–overload states resulting in ischaemia and fibrosis; (2) data from animal studies have shown that pro–fibrotic pathways are activated to a larger extent in pressure–overload states compared to volume–overload states; (3) the predominant pathology in MR may be extracellular volume loss, rather than excessive collagen deposition secondary to activation of Kallikrin–Kinin system, and thereby, of bradykinin, which increases MMP activity, causing loss of collagen, and LV dysfunction, as shown in an animal model.^29^ The predominance of degradation over synthesis results in loss and disruption of the myocardial collagen scaffold and an associated decline in matrix tensile strength, resulting in ventricular dilatation, systolic dysfunction and ultimately death.^14^

In this study, patients with CRMR had increased collagen degradation, as suggested by increase in MMP activity and normal levels of TIMPs and markers of collagen synthesis. This finding supports the lack of myocardial fibrosis observed in our study. These findings differ from the study by Banerjee et al. in 30 patients with CRMR, where they found an increased level of biomarkers of synthesis and degradation.^13^ The discrepancy may be explained by: younger patients than in this study (mean age 29.6 ± 2 years), possible ongoing rheumatic activity, and the inclusion of patients with atrial fibrillation, therefore resulting in increased biomarker levels.^13^

The use of anti–remodelling therapy was similar in our study to that of Banerjee et al.^13^ Thirty to 40% of their patients were on anti–remodelling therapy with spironolactone and ACE inhibitors, respectively, and 10% were on beta–blockers. In their study, only biopsies of the leaflets were performed, not the LV to assess the absence or presence of fibrosis. Furthermore, they reported increased thickness of the leaflets and collagen deposition in eight patients who underwent surgery. It is unclear, however, as to whether the primary lesion was mitral stenosis or MR in this subset of patients.

Moreover, there was increased MMP activity in their MR patients compared to mitral stenosis, as well as increased MMP–to–TIMP ratio. They acknowledge that the elevation in PIP levels was lower than anticipated in their MR cohort, and that markers of collagen degradation exceeded markers of synthesis in their patients with CRMR.

The main limitation of this study was the small sample size. A larger sample size would have reduced the probability of chance accounting for the absence or presence of fibrosis. A study with a larger sample size with isolated MR and one with co–morbidities and MR may be required to account for the finding of fibrosis secondary to isolated MR. T1 mapping was not used to exclude the presence of microscopic fibrosis and LV biopsies were not performed.

## Conclusion

The occurrence of LV fibrosis by LGE imaging was low in CRMR patients. This finding corroborates the increased level of biomarkers of collagen degradation and normal levels of biomarkers of collagen synthesis. These findings may have implications in terms of therapy. Earlier surgical referral may be of benefit before dissolution of the myocardial scaffold and irreversible myocardial damage ensues, with resultant poor postoperative LV function.
